# Bis(di-2-pyridyl­amine-κ^2^ *N* ^2^,*N* ^2’^)platinum(II) dibromide monohydrate

**DOI:** 10.1107/S1600536812013062

**Published:** 2012-03-31

**Authors:** Kwang Ha

**Affiliations:** a School of Applied Chemical Engineering, The Research Institute of Catalysis, Chonnam National University, Gwangju 500-757, Republic of Korea

## Abstract

The asymmetric unit of the title compound, [Pt(C_10_H_9_N_3_)_2_]Br_2_·H_2_O, contains two crystallographically independent half-mol­ecules of the cationic Pt^II^ complex, two Br^−^ anions and a lattice water mol­ecule; an inversion centre is located at the centroid of each complex. Each Pt^II^ ion is four-coordinated in an essentially square-planar environment by four pyridine N atoms derived from the two chelating di-2-pyridyl­amine (dpa) ligands, and the PtN_4_ unit is exactly planar. The chelate ring formed by the dpa ligand displays a boat conformation, with dihedral angles between the pyridine rings of 35.9 (2) and 41.0 (2)°. The complex cations, Br^−^ anions and solvent water mol­ecules are linked by O—H⋯Br, N—H⋯Br, C—H⋯O and C—H⋯Br hydrogen bonds, forming a three-dimensional network.

## Related literature

For the crystal structures of the related Pd^II^ and Pt^II^ complexes, see: Živković *et al.* (2007 ▶); Antonioli *et al.* (2008 ▶); Guney *et al.* (2010 ▶).
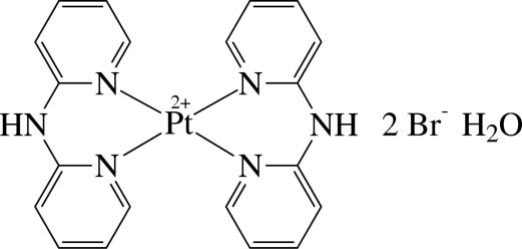


## Experimental

### 

#### Crystal data

[Pt(C_10_H_9_N_3_)_2_]Br_2_·H_2_O
*M* *_r_* = 715.33
Triclinic, 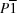

*a* = 9.7870 (9) Å
*b* = 11.059 (1) Å
*c* = 12.1151 (12) Å
α = 109.448 (2)°
β = 104.538 (2)°
γ = 107.980 (2)°
*V* = 1080.70 (18) Å^3^
*Z* = 2
Mo *K*α radiation
μ = 10.21 mm^−1^
*T* = 200 K
0.27 × 0.17 × 0.12 mm

#### Data collection

Bruker SMART 1000 CCD diffractometer
Absorption correction: multi-scan (*SADABS*; Bruker, 2000 ▶) *T*_min_ = 0.745, *T*_max_ = 1.000
6721 measured reflections
4109 independent reflections
3411 reflections with *I* > 2σ(*I*)
*R*_int_ = 0.024

#### Refinement

*R*[*F* ^2^ > 2σ(*F* ^2^)] = 0.029
*wR*(*F* ^2^) = 0.069
*S* = 1.05
4109 reflections
274 parameters
H-atom parameters constrained
Δρ_max_ = 1.43 e Å^−3^
Δρ_min_ = −1.21 e Å^−3^

### 

Data collection: *SMART* (Bruker, 2000 ▶); cell refinement: *SAINT* (Bruker, 2000 ▶); data reduction: *SAINT*; program(s) used to solve structure: *SHELXS97* (Sheldrick, 2008 ▶); program(s) used to refine structure: *SHELXL97* (Sheldrick, 2008 ▶); molecular graphics: *ORTEP-3* (Farrugia, 1997 ▶) and *PLATON* (Spek, 2009 ▶); software used to prepare material for publication: *SHELXL97*.

## Supplementary Material

Crystal structure: contains datablock(s) global. DOI: 10.1107/S1600536812013062/bq2347sup1.cif

Additional supplementary materials:  crystallographic information; 3D view; checkCIF report

## Figures and Tables

**Table:** 

Pt1—N1	2.013 (5)
Pt1—N3	2.030 (5)
Pt2—N6	2.014 (4)
Pt2—N4	2.024 (4)

**Table:** 

N1—Pt1—N3	87.10 (19)
N6—Pt2—N4	86.65 (17)

**Table 2:** Hydrogen-bond geometry (Å, °)

*D*—H⋯*A*	*D*—H	H⋯*A*	*D*⋯*A*	*D*—H⋯*A*
O1—H1*A*⋯Br1^i^	0.84	2.58	3.399 (6)	166
O1—H1*B*⋯Br1^ii^	0.84	2.54	3.374 (5)	171
N2—H2*N*⋯Br1^iii^	0.92	2.38	3.289 (4)	171
N5—H5*N*⋯Br2	0.92	2.35	3.267 (4)	174
C2—H2⋯O1^iv^	0.95	2.58	3.302 (8)	133
C11—H11⋯Br2^ii^	0.95	2.87	3.635 (6)	138
C13—H13⋯Br2^v^	0.95	2.76	3.712 (6)	177
C20—H20⋯O1^vi^	0.95	2.56	3.464 (8)	160

Symmetry codes: (i) 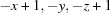; (ii) 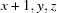; (iii) 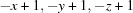; (iv) 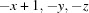; (v) 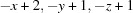; (vi) 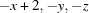.

**Fig. 1.**
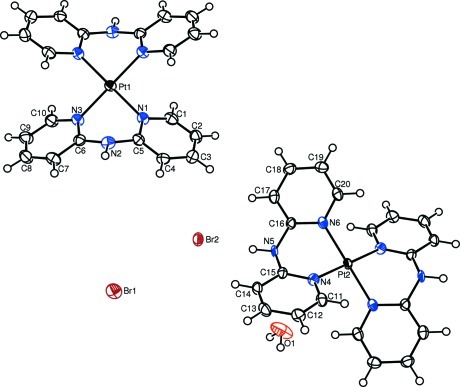
A structure detail of the title compound, with displacement ellipsoids drawn at the 50% probability level for non-H atoms. Unlabelled atoms are generated by the application of the inversion centers.

**Fig. 2.**
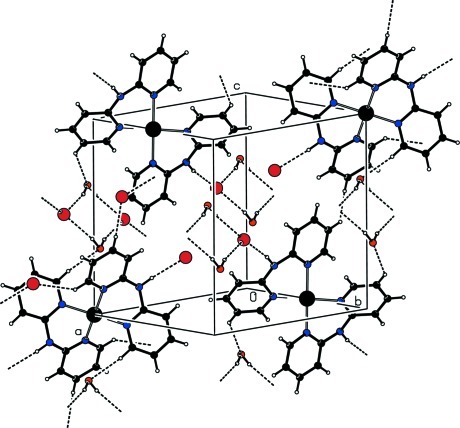
A view of the unit-cell contents of the title compound. Hydrogen-bond interactions are drawn with dashed lines.

## supplementary crystallographic information

### Comment

The title compound, [Pt(dpa)_2_]Br_2_.H_2_O (dpa = di-2-pyridylamine), was unexpected obtained from the reaction of K_2_PtBr_6_ with dpa. It seems that the Pt^IV^ ion reduced to the Pt^II^ ion in the reaction. Crystal structures of the related cationic Pd^II^ and Pt^II^ complexes, such as [Pd(dpa)_2_](*X*)_2_ (*X* = Cl or PF_6_) (Živković *et al.*, 2007; Antonioli *et al.*, 2008) and [*M*(dpa)_2_](sac)_2_ (*M* = Pd or Pt; sac = saccharinate) (Guney *et al.*, 2010), have been investigated previously.

The asymmetric unit contains two crystallographically independent half-molecules of the cationic Pt^II^ complex, two Br^-^ anions and a lattice water molecule; an inversion centre is located at the centroid of each complex (Fig. 1). The two complexes are chemically identical, but slightly different in geometry. The Pt^II^ ion in each complex is four-coordinated in an essentially square-planar environment by four pyridine N atoms derived from the two chelating dpa ligands, and the PtN_4_ unit is exactly planar. The dpa ligands display a boat conformation with dihedral angles between the least-squares planes of the two pyridine rings of 35.9 (2)° in complex with Pt1 and 41.0 (2)° in complex with Pt2. The Pt—N bond lengths are nearly equivalent [Pt—N: 2.013 (5)–2.030 (5) Å] (Table 1). The complex cations, Br^-^ anions and solvent water molecules are linked by intermolecular O—H···Br, N—H···Br, C—H···O and C—H···Br hydrogen bonds, forming a three-dimensional network (Fig. 2 and Table 2). The complex cations are stacked into columns along the *a* axis and show a number of intermolecular π-π interactions between the pyridine rings, with a shortest ring centroid-centroid distance of 3.951 (4) Å.

### Experimental

To a solution of K_2_PtBr_6_ (0.1016 g, 0.135 mmol) in H_2_O (10 ml) and MeOH (10 ml) was added di-2-pyridylamine (0.0479 g, 0.280 mmol) and stirred for 7 h at room temperature. The formed precipitate was separated by filtration and washed with H_2_O and MeOH, and dried at 50 °C, to give an orange powder (0.0450 g). Crystals suitable for X-ray analysis were obtained by slow evaporation from an *N,N*-dimethylformamide (DMF) solution at 60 °C.

### Refinement

Carbon-bound H atoms were positioned geometrically and allowed to ride on their respective parent atoms: C—H = 0.95 Å with *U*_iso_(H) = 1.2*U*_eq_(C). Nitrogen- and oxygen-bound H atoms were located from the difference Fourier map then allowed to ride on their parent atoms in the final cycles of refinement with N—H = 0.92 Å, O—H = 0.84 Å and *U*_iso_(H) = 1.5 *U*_eq_(N, O). The highest peak (1.43 e Å^-3^) and the deepest hole (-1.21 e Å^-3^) in the difference Fourier map are located 1.64 Å and 0.83 Å from the atoms Br1 and Pt1, respectively.

### Crystal data

**Table:**

[Pt(C_10_H_9_N_3_)_2_]Br_2_·H_2_O	*Z* = 2
*M**_r_* = 715.33	*F*(000) = 676
Triclinic, *P*1	*D*_x_ = 2.198 Mg m^−^^3^
Hall symbol: -P 1	Mo *K*α radiation, λ = 0.71073 Å
*a* = 9.7870 (9) Å	Cell parameters from 4253 reflections
*b* = 11.059 (1) Å	θ = 2.2–26.0°
*c* = 12.1151 (12) Å	µ = 10.21 mm^−^^1^
α = 109.448 (2)°	*T* = 200 K
β = 104.538 (2)°	Block, yellow
γ = 107.980 (2)°	0.27 × 0.17 × 0.12 mm
*V* = 1080.70 (18) Å^3^	

### Data collection

**Table:**

Bruker SMART 1000 CCD diffractometer	4109 independent reflections
Radiation source: fine-focus sealed tube	3411 reflections with *I* > 2σ(*I*)
Graphite monochromator	*R*_int_ = 0.024
φ and ω scans	θ_max_ = 26.0°, θ_min_ = 1.9°
Absorption correction: multi-scan (*SADABS*; Bruker, 2000)	*h* = −11→12
*T*_min_ = 0.745, *T*_max_ = 1.000	*k* = −13→13
6721 measured reflections	*l* = −14→11

### Refinement

**Table:**

Refinement on *F*^2^	Primary atom site location: structure-invariant direct methods
Least-squares matrix: full	Secondary atom site location: difference Fourier map
*R*[*F*^2^ > 2σ(*F*^2^)] = 0.029	Hydrogen site location: inferred from neighbouring sites
*wR*(*F*^2^) = 0.069	H-atom parameters constrained
*S* = 1.05	*w* = 1/[σ^2^(*F*_o_^2^) + (0.0253*P*)^2^ + 0.3123*P*] where *P* = (*F*_o_^2^ + 2*F*_c_^2^)/3
4109 reflections	(Δ/σ)_max_ < 0.001
274 parameters	Δρ_max_ = 1.43 e Å^−^^3^
0 restraints	Δρ_min_ = −1.21 e Å^−^^3^

### Special details

**Table:**

Geometry. All e.s.d.'s (except the e.s.d. in the dihedral angle between two l.s. planes) are estimated using the full covariance matrix. The cell e.s.d.'s are taken into account individually in the estimation of e.s.d.'s in distances, angles and torsion angles; correlations between e.s.d.'s in cell parameters are only used when they are defined by crystal symmetry. An approximate (isotropic) treatment of cell e.s.d.'s is used for estimating e.s.d.'s involving l.s. planes.
Refinement. Refinement of *F*^2^ against ALL reflections. The weighted *R*-factor *wR* and goodness of fit *S* are based on *F*^2^, conventional *R*-factors *R* are based on *F*, with *F* set to zero for negative *F*^2^. The threshold expression of *F*^2^ > σ(*F*^2^) is used only for calculating *R*-factors(gt) *etc*. and is not relevant to the choice of reflections for refinement. *R*-factors based on *F*^2^ are statistically about twice as large as those based on *F*, and *R*- factors based on ALL data will be even larger.

### Fractional atomic coordinates and isotropic or equivalent isotropic displacement parameters (Å^2^)

**Table:**

	*x*	*y*	*z*	*U*_iso_*/*U*_eq_	
Pt1	0.0000	0.5000	0.0000	0.02139 (9)	
N1	0.1711 (5)	0.4350 (5)	0.0277 (4)	0.0240 (11)	
N2	0.3215 (5)	0.6364 (5)	0.2257 (5)	0.0269 (11)	
H2N	0.4183	0.7004	0.2890	0.040*	
N3	0.0561 (5)	0.5768 (5)	0.1905 (4)	0.0246 (11)	
C1	0.1575 (7)	0.3112 (6)	−0.0598 (6)	0.0298 (14)	
H1	0.0581	0.2463	−0.1264	0.036*	
C2	0.2805 (7)	0.2766 (6)	−0.0557 (6)	0.0312 (15)	
H2	0.2676	0.1907	−0.1194	0.037*	
C3	0.4250 (8)	0.3699 (7)	0.0437 (6)	0.0344 (15)	
H3	0.5136	0.3506	0.0470	0.041*	
C4	0.4381 (7)	0.4897 (6)	0.1368 (6)	0.0291 (14)	
H4	0.5354	0.5527	0.2067	0.035*	
C5	0.3093 (7)	0.5189 (6)	0.1287 (5)	0.0243 (13)	
C6	0.2076 (7)	0.6479 (6)	0.2715 (5)	0.0244 (13)	
C7	0.2502 (7)	0.7317 (6)	0.4000 (6)	0.0298 (14)	
H7	0.3575	0.7840	0.4560	0.036*	
C8	0.1366 (8)	0.7384 (7)	0.4452 (6)	0.0372 (16)	
H8	0.1643	0.7999	0.5317	0.045*	
C9	−0.0200 (7)	0.6543 (6)	0.3636 (6)	0.0329 (15)	
H9	−0.1002	0.6529	0.3946	0.039*	
C10	−0.0555 (7)	0.5748 (6)	0.2397 (6)	0.0302 (14)	
H10	−0.1621	0.5150	0.1845	0.036*	
Pt2	1.0000	0.0000	0.0000	0.01798 (9)	
N4	1.0831 (5)	0.1819 (4)	0.1611 (4)	0.0220 (11)	
N5	0.8237 (5)	0.1494 (5)	0.1336 (4)	0.0235 (11)	
H5N	0.7698	0.1868	0.1763	0.035*	
N6	0.8306 (5)	0.0536 (4)	−0.0701 (4)	0.0203 (10)	
C11	1.2399 (7)	0.2590 (6)	0.2295 (5)	0.0245 (13)	
H11	1.3090	0.2334	0.1944	0.029*	
C12	1.3011 (7)	0.3710 (6)	0.3461 (5)	0.0267 (14)	
H12	1.4110	0.4235	0.3918	0.032*	
C13	1.1976 (7)	0.4073 (6)	0.3976 (6)	0.0295 (14)	
H13	1.2371	0.4832	0.4803	0.035*	
C14	1.0396 (7)	0.3330 (5)	0.3281 (5)	0.0247 (13)	
H14	0.9686	0.3570	0.3617	0.030*	
C15	0.9845 (6)	0.2211 (5)	0.2068 (5)	0.0203 (12)	
C16	0.7593 (6)	0.1050 (5)	0.0029 (5)	0.0208 (12)	
C17	0.6218 (7)	0.1153 (6)	−0.0491 (6)	0.0260 (13)	
H17	0.5704	0.1472	0.0033	0.031*	
C18	0.5612 (7)	0.0790 (6)	−0.1767 (6)	0.0288 (14)	
H18	0.4653	0.0821	−0.2139	0.035*	
C19	0.6406 (7)	0.0379 (6)	−0.2507 (6)	0.0272 (14)	
H19	0.6047	0.0192	−0.3378	0.033*	
C20	0.7725 (7)	0.0247 (5)	−0.1956 (5)	0.0243 (13)	
H20	0.8258	−0.0057	−0.2469	0.029*	
Br1	0.31515 (7)	0.15218 (7)	0.57090 (7)	0.04448 (19)	
Br2	0.63618 (7)	0.30058 (6)	0.27528 (6)	0.02942 (15)	
O1	0.9598 (6)	0.0001 (6)	0.3324 (5)	0.0680 (17)	
H1A	0.9056	−0.0343	0.3676	0.102*	
H1B	1.0433	0.0410	0.3975	0.102*	

### Atomic displacement parameters (Å^2^)

**Table:**

	*U*^11^	*U*^22^	*U*^33^	*U*^12^	*U*^13^	*U*^23^
Pt1	0.01682 (16)	0.02405 (17)	0.01970 (17)	0.00604 (13)	0.00550 (13)	0.00954 (14)
N1	0.020 (2)	0.023 (2)	0.021 (3)	0.005 (2)	0.006 (2)	0.007 (2)
N2	0.018 (3)	0.024 (2)	0.029 (3)	0.003 (2)	0.004 (2)	0.010 (2)
N3	0.019 (3)	0.027 (3)	0.022 (3)	0.006 (2)	0.007 (2)	0.009 (2)
C1	0.033 (4)	0.027 (3)	0.023 (3)	0.008 (3)	0.008 (3)	0.010 (3)
C2	0.037 (4)	0.029 (3)	0.026 (3)	0.015 (3)	0.013 (3)	0.009 (3)
C3	0.039 (4)	0.041 (4)	0.035 (4)	0.025 (3)	0.019 (3)	0.020 (3)
C4	0.028 (3)	0.037 (3)	0.024 (3)	0.016 (3)	0.008 (3)	0.015 (3)
C5	0.024 (3)	0.024 (3)	0.020 (3)	0.008 (2)	0.007 (2)	0.008 (3)
C6	0.028 (3)	0.020 (3)	0.025 (3)	0.011 (2)	0.009 (3)	0.011 (3)
C7	0.028 (3)	0.025 (3)	0.023 (3)	0.002 (3)	0.005 (3)	0.008 (3)
C8	0.054 (5)	0.036 (4)	0.025 (3)	0.022 (3)	0.017 (3)	0.014 (3)
C9	0.031 (4)	0.043 (4)	0.031 (3)	0.016 (3)	0.016 (3)	0.021 (3)
C10	0.026 (3)	0.036 (3)	0.029 (3)	0.013 (3)	0.011 (3)	0.015 (3)
Pt2	0.01701 (16)	0.01930 (16)	0.01565 (16)	0.00789 (12)	0.00733 (12)	0.00487 (13)
N4	0.028 (3)	0.014 (2)	0.023 (3)	0.009 (2)	0.009 (2)	0.006 (2)
N5	0.026 (3)	0.031 (3)	0.014 (2)	0.018 (2)	0.011 (2)	0.003 (2)
N6	0.024 (3)	0.015 (2)	0.018 (2)	0.0069 (19)	0.009 (2)	0.003 (2)
C11	0.028 (3)	0.024 (3)	0.026 (3)	0.011 (3)	0.016 (3)	0.012 (3)
C12	0.020 (3)	0.024 (3)	0.021 (3)	0.003 (2)	0.003 (3)	0.004 (3)
C13	0.036 (4)	0.025 (3)	0.019 (3)	0.011 (3)	0.009 (3)	0.003 (3)
C14	0.035 (3)	0.023 (3)	0.023 (3)	0.017 (3)	0.015 (3)	0.009 (3)
C15	0.022 (3)	0.020 (3)	0.020 (3)	0.011 (2)	0.009 (2)	0.007 (2)
C16	0.021 (3)	0.016 (3)	0.019 (3)	0.009 (2)	0.006 (2)	0.002 (2)
C17	0.031 (3)	0.021 (3)	0.027 (3)	0.013 (3)	0.015 (3)	0.008 (3)
C18	0.026 (3)	0.030 (3)	0.026 (3)	0.015 (3)	0.005 (3)	0.010 (3)
C19	0.032 (3)	0.025 (3)	0.024 (3)	0.011 (3)	0.008 (3)	0.013 (3)
C20	0.030 (3)	0.019 (3)	0.021 (3)	0.009 (2)	0.012 (3)	0.006 (3)
Br1	0.0234 (3)	0.0497 (4)	0.0373 (4)	0.0048 (3)	0.0039 (3)	0.0099 (3)
Br2	0.0253 (3)	0.0343 (3)	0.0247 (3)	0.0157 (3)	0.0101 (3)	0.0058 (3)
O1	0.051 (4)	0.093 (4)	0.036 (3)	0.007 (3)	0.009 (3)	0.032 (3)

### Geometric parameters (Å, º)

**Table:**

Pt1—N1^i^	2.013 (5)	Pt2—N6^ii^	2.014 (4)
Pt1—N1	2.013 (5)	Pt2—N4^ii^	2.024 (4)
Pt1—N3^i^	2.030 (5)	Pt2—N4	2.024 (4)
Pt1—N3	2.030 (5)	N4—C15	1.339 (7)
N1—C5	1.351 (7)	N4—C11	1.361 (7)
N1—C1	1.366 (7)	N5—C16	1.391 (7)
N2—C5	1.379 (7)	N5—C15	1.397 (7)
N2—C6	1.384 (7)	N5—H5N	0.9200
N2—H2N	0.9200	N6—C16	1.346 (7)
N3—C6	1.348 (7)	N6—C20	1.367 (7)
N3—C10	1.367 (8)	C11—C12	1.356 (7)
C1—C2	1.366 (8)	C11—H11	0.9500
C1—H1	0.9500	C12—C13	1.406 (8)
C2—C3	1.389 (8)	C12—H12	0.9500
C2—H2	0.9500	C13—C14	1.370 (8)
C3—C4	1.368 (8)	C13—H13	0.9500
C3—H3	0.9500	C14—C15	1.401 (7)
C4—C5	1.384 (8)	C14—H14	0.9500
C4—H4	0.9500	C16—C17	1.392 (7)
C6—C7	1.390 (8)	C17—C18	1.372 (8)
C7—C8	1.369 (9)	C17—H17	0.9500
C7—H7	0.9500	C18—C19	1.383 (8)
C8—C9	1.392 (9)	C18—H18	0.9500
C8—H8	0.9500	C19—C20	1.371 (8)
C9—C10	1.348 (8)	C19—H19	0.9500
C9—H9	0.9500	C20—H20	0.9500
C10—H10	0.9500	O1—H1A	0.8400
Pt2—N6	2.014 (4)	O1—H1B	0.8400
			
N1^i^—Pt1—N1	180.000 (1)	N6—Pt2—N4^ii^	93.35 (17)
N1^i^—Pt1—N3^i^	87.10 (19)	N6^ii^—Pt2—N4^ii^	86.65 (17)
N1—Pt1—N3^i^	92.90 (19)	N6—Pt2—N4	86.65 (17)
N1^i^—Pt1—N3	92.90 (19)	N6^ii^—Pt2—N4	93.35 (17)
N1—Pt1—N3	87.10 (19)	N4^ii^—Pt2—N4	180.0 (3)
N3^i^—Pt1—N3	180.00 (11)	C15—N4—C11	119.4 (5)
C5—N1—C1	117.5 (5)	C15—N4—Pt2	120.3 (4)
C5—N1—Pt1	120.7 (4)	C11—N4—Pt2	120.2 (4)
C1—N1—Pt1	121.6 (4)	C16—N5—C15	123.2 (5)
C5—N2—C6	127.0 (5)	C16—N5—H5N	113.7
C5—N2—H2N	119.1	C15—N5—H5N	111.0
C6—N2—H2N	109.0	C16—N6—C20	117.8 (5)
C6—N3—C10	118.5 (5)	C16—N6—Pt2	120.3 (4)
C6—N3—Pt1	119.5 (4)	C20—N6—Pt2	121.6 (4)
C10—N3—Pt1	121.4 (4)	C12—C11—N4	122.4 (5)
C2—C1—N1	122.9 (5)	C12—C11—H11	118.8
C2—C1—H1	118.5	N4—C11—H11	118.8
N1—C1—H1	118.5	C11—C12—C13	118.3 (5)
C1—C2—C3	118.5 (6)	C11—C12—H12	120.9
C1—C2—H2	120.7	C13—C12—H12	120.9
C3—C2—H2	120.7	C14—C13—C12	119.8 (5)
C4—C3—C2	119.3 (6)	C14—C13—H13	120.1
C4—C3—H3	120.4	C12—C13—H13	120.1
C2—C3—H3	120.4	C13—C14—C15	118.9 (5)
C3—C4—C5	119.8 (6)	C13—C14—H14	120.5
C3—C4—H4	120.1	C15—C14—H14	120.5
C5—C4—H4	120.1	N4—C15—N5	120.0 (5)
N1—C5—N2	118.9 (5)	N4—C15—C14	121.0 (5)
N1—C5—C4	121.6 (5)	N5—C15—C14	118.9 (5)
N2—C5—C4	119.5 (5)	N6—C16—N5	119.7 (5)
N3—C6—N2	119.4 (5)	N6—C16—C17	121.6 (5)
N3—C6—C7	120.7 (6)	N5—C16—C17	118.7 (5)
N2—C6—C7	119.9 (5)	C18—C17—C16	119.5 (6)
C8—C7—C6	119.5 (6)	C18—C17—H17	120.3
C8—C7—H7	120.3	C16—C17—H17	120.3
C6—C7—H7	120.3	C17—C18—C19	119.5 (5)
C7—C8—C9	119.5 (6)	C17—C18—H18	120.2
C7—C8—H8	120.2	C19—C18—H18	120.2
C9—C8—H8	120.2	C20—C19—C18	118.5 (6)
C10—C9—C8	118.7 (6)	C20—C19—H19	120.8
C10—C9—H9	120.7	C18—C19—H19	120.8
C8—C9—H9	120.7	N6—C20—C19	122.8 (6)
C9—C10—N3	122.5 (6)	N6—C20—H20	118.6
C9—C10—H10	118.7	C19—C20—H20	118.6
N3—C10—H10	118.7	H1A—O1—H1B	93.7
N6—Pt2—N6^ii^	180.0 (2)		
			
N3^i^—Pt1—N1—C5	138.4 (5)	N6—Pt2—N4—C15	41.4 (4)
N3—Pt1—N1—C5	−41.6 (5)	N6^ii^—Pt2—N4—C15	−138.6 (4)
N3^i^—Pt1—N1—C1	−35.8 (5)	N6—Pt2—N4—C11	−144.2 (4)
N3—Pt1—N1—C1	144.2 (5)	N6^ii^—Pt2—N4—C11	35.8 (4)
N1^i^—Pt1—N3—C6	−136.6 (4)	N4^ii^—Pt2—N6—C16	136.8 (4)
N1—Pt1—N3—C6	43.4 (4)	N4—Pt2—N6—C16	−43.2 (4)
N1^i^—Pt1—N3—C10	34.7 (5)	N4^ii^—Pt2—N6—C20	−35.8 (4)
N1—Pt1—N3—C10	−145.3 (5)	N4—Pt2—N6—C20	144.2 (4)
C5—N1—C1—C2	−6.6 (9)	C15—N4—C11—C12	3.2 (8)
Pt1—N1—C1—C2	167.8 (5)	Pt2—N4—C11—C12	−171.2 (4)
N1—C1—C2—C3	1.7 (10)	N4—C11—C12—C13	0.4 (9)
C1—C2—C3—C4	2.7 (9)	C11—C12—C13—C14	−2.2 (9)
C2—C3—C4—C5	−2.1 (9)	C12—C13—C14—C15	0.4 (9)
C1—N1—C5—N2	−172.9 (5)	C11—N4—C15—N5	175.4 (5)
Pt1—N1—C5—N2	12.7 (7)	Pt2—N4—C15—N5	−10.2 (7)
C1—N1—C5—C4	7.2 (8)	C11—N4—C15—C14	−5.1 (8)
Pt1—N1—C5—C4	−167.2 (5)	Pt2—N4—C15—C14	169.4 (4)
C6—N2—C5—N1	36.1 (8)	C16—N5—C15—N4	−40.2 (7)
C6—N2—C5—C4	−144.0 (6)	C16—N5—C15—C14	140.2 (5)
C3—C4—C5—N1	−3.0 (9)	C13—C14—C15—N4	3.3 (8)
C3—C4—C5—N2	177.1 (6)	C13—C14—C15—N5	−177.2 (5)
C10—N3—C6—N2	171.8 (5)	C20—N6—C16—N5	−173.5 (5)
Pt1—N3—C6—N2	−16.6 (7)	Pt2—N6—C16—N5	13.6 (7)
C10—N3—C6—C7	−8.0 (8)	C20—N6—C16—C17	6.4 (8)
Pt1—N3—C6—C7	163.6 (4)	Pt2—N6—C16—C17	−166.5 (4)
C5—N2—C6—N3	−33.6 (8)	C15—N5—C16—N6	38.2 (7)
C5—N2—C6—C7	146.2 (6)	C15—N5—C16—C17	−141.6 (5)
N3—C6—C7—C8	2.1 (9)	N6—C16—C17—C18	−3.3 (9)
N2—C6—C7—C8	−177.7 (5)	N5—C16—C17—C18	176.6 (5)
C6—C7—C8—C9	4.0 (9)	C16—C17—C18—C19	−2.4 (9)
C7—C8—C9—C10	−4.1 (9)	C17—C18—C19—C20	4.7 (9)
C8—C9—C10—N3	−2.0 (10)	C16—N6—C20—C19	−4.0 (8)
C6—N3—C10—C9	8.0 (9)	Pt2—N6—C20—C19	168.8 (4)
Pt1—N3—C10—C9	−163.3 (5)	C18—C19—C20—N6	−1.5 (9)

Symmetry codes: (i) −*x*, −*y*+1, −*z*; (ii) −*x*+2, −*y*, −*z*.

### Hydrogen-bond geometry (Å, º)

**Table:**

*D*—H···*A*	*D*—H	H···*A*	*D*···*A*	*D*—H···*A*
O1—H1*A*···Br1^iii^	0.84	2.58	3.399 (6)	166
O1—H1*B*···Br1^iv^	0.84	2.54	3.374 (5)	171
N2—H2*N*···Br1^v^	0.92	2.38	3.289 (4)	171
N5—H5*N*···Br2	0.92	2.35	3.267 (4)	174
C2—H2···O1^vi^	0.95	2.58	3.302 (8)	133
C11—H11···Br2^iv^	0.95	2.87	3.635 (6)	138
C13—H13···Br2^vii^	0.95	2.76	3.712 (6)	177
C20—H20···O1^ii^	0.95	2.56	3.464 (8)	160

Symmetry codes: (ii) −*x*+2, −*y*, −*z*; (iii) −*x*+1, −*y*, −*z*+1; (iv) *x*+1, *y*, *z*; (v) −*x*+1, −*y*+1, −*z*+1; (vi) −*x*+1, −*y*, −*z*; (vii) −*x*+2, −*y*+1, −*z*+1.
